# Retraction: A mathematical model of blood flow in a stenosed artery with post-stenotic dilatation and a forced field

**DOI:** 10.1371/journal.pone.0362616

**Published:** 2026-11-12

**Authors:** The PLOS One Editors

The *PLOS One* Editors retract this article [1] due to concerns about potential manipulation of the publication process, peer review integrity, and authorship. These concerns call into question the validity and provenance of the reported results. We regret that the issues were not identified prior to the article’s publication.

MD, GS, MRE, and UB did not agree with the retraction. RS, WJ, SG, and RC either did not respond directly or could not be reached.
